# Patients’ and relatives’ perspectives on best possible care in the context of developing a multidisciplinary center for endometriosis and adenomyosis: findings from a national survey

**DOI:** 10.1186/s12905-022-01798-8

**Published:** 2022-06-10

**Authors:** Marianne Omtvedt, Elisabeth Bean, Kirsten Hald, Elisabeth Raasholm Larby, Guri B. Majak, Tina Tellum

**Affiliations:** 1 Department of Gynecology, Oslo University Hospital, Nydalen, P. O. BOX 4950, 0424 Oslo, Norway; 2 Institute of Clinical Medicine, Faculty of Medicine, University of Oslo, Oslo, Norway; 3 Institute for Women’s Health, University College London Hospitals, London, UK; 4 Norwegian Patient’s Endometriosis Society, Patient and Leader of the Society, Halden, Norway

**Keywords:** Patient-centeredness, Quality of care, Women’s health, Endometriosis, Adenomyosis, Multidisciplinary care, Centralized endometriosis center

## Abstract

**Background:**

Endometriosis and adenomyosis are common benign conditions compromising both physical and psychological health, with a negative impact on quality of life. This survey aimed to establish what the users’ perspectives are on best possible care in the context of developing a multidisciplinary center for endometriosis and adenomyosis in Norway.

**Methods:**

An electronic questionnaire was developed in collaboration between the Norwegian Patient’s Endometriosis Society (NPES) and gynecologists with special interest in endometriosis and adenomyosis. The questionnaire was distributed digitally to the members of NPES in May 2021.

**Results:**

938 participants answered the questionnaire. Better patient information, long term therapeutic plans and integration of their partners into their care were the main concerns. Multidisciplinary care was a key issue for the majority, with (n = 775) 89% stating a need for a consultation with a psychologist, (n = 744) 86% at least one consultation with a nutritionist, (n = 733) 85% a physiotherapist, and (n = 676) 78% needing a sex therapist and (n = 935) 99,7% consider research and (n = 934) 99,8% consider quality assurance initiated by the endometriosis center to be important. The qualitative analysis of free text answers revealed a great need for updated and easily accessible information, meeting competent health care professionals and being taken seriously/listened to.

**Conclusions:**

This survey shows similar perceptions and a high level of agreement regarding their needs amongst people with endometriosis and/or adenomyosis. This survey supports recommendations by the experts that endometriosis/adenomyosis care should be centralized in specialized, multidisciplinary centers. The results of the present work will be valuable for the future planning and development of a multidisciplinary endometriosis center.

**Supplementary Information:**

The online version contains supplementary material available at 10.1186/s12905-022-01798-8.

## Background

Endometriosis is characterized as a chronic inflammatory disease defined by the presence of endometrial-like tissue outside the uterus [1]. The prevalence varies in different studies, but ranges from 6 to 10% [2]. Endometriosis presents with various symptoms including dysmenorrhea, ovulation pain, deep dyspareunia, chronic pelvic pain, infertility and chronic fatigue [1]. Adenomyosis is a similar condition and is frequently associated with endometriosis [3]. It is characterized by the infiltration of endometrial tissue into the myometrium of the uterus [4]. Adenomyosis also leads to symptoms of dysmenorrhea, menorrhagia and chronic pelvic pain [5], and contributes to infertility and adverse pregnancy outcomes [6].

Endometriosis and adenomyosis can be challenging to diagnose and manage, both with regards to symptom control and fertility related issues. As a result, there is significant delay between symptom onset and a definitive diagnosis of endometriosis, and it is suspected to be even longer for adenomyosis [7–9]. Both conditions are shown to have a negative impact on quality of life and can lead to depression and fatigue [10]. Endometriosis causes a substantial economic burden, mostly due to loss of work productivity, comparable to other chronic diseases like diabetes and rheumatoid arthritis [11–13]. This is an important aspect, since almost all patients with endometriosis are of a working age. The impact of adenomyosis on women’s health has not been adequately studied [5].

Since 2005, experts have recommended that patients with advanced endometriosis should be referred to a center that offers multidisciplinary management of endometriosis care [1]. “The challenge of endometriosis” and the need for multidisciplinary centers was also highlighted by D’Hooge and Hummelshoj, emphasizing that women with endometriosis should receive “consistent, evidence-based care, ensuring excellence, continuity of care, multi-disciplinarity, research, training and cost-effectiveness” [14].

In addition to multidisciplinary care, an important dimension of health care quality is patient-centered care [15], which is defined as being “respectful of and responsive to individual patient preferences and needs” and “guided by patients’ values” [16]. In a review, Dancet et al. [17] showed that women with endometriosis need more than effective and safe care, but also special attention to respecting patients, believing patients and timely diagnosis of endometriosis.

Several endometriosis centers have been established all over the world. In Scandinavia, Denmark was the first country to centralize the treatment of advanced endometriosis over 20 years ago. Since then, both Sweden and Denmark have established several endometriosis centers. Although Norway has a similar health care system and patient demographics, and despite increasing demand from patients and attempts by doctors, a formal endometriosis center has not yet been established.

When trying to organize the best possible care for women with endometriosis and adenomyosis, without additional funding, it is important to prioritize the most essential needs. The aim of this survey is to find out what patients and relatives consider to be the best possible care in terms of multidisciplinary endometriosis and adenomyosis management and potential development of an endometriosis center, to prioritize resources towards the most needed aspects.

In this article, we use the terms "woman" and "women", but it is important to note that endometriosis and adenomyosis can affect all assigned female at birth (not only those who identify as women).

## Methods

### Study design

This is a prospective, questionnaire based, mixed method survey. Ethics approval by the National research ethics system was deemed unnecessary according to the Norwegian Act on medical and health research [18], and waived by the institutional review board (IRB) and the data protection officer (DPO) at Oslo University Hospital. An electronic questionnaire was developed in close collaboration between the Norwegian Patient’s Endometriosis Society (NPES) and gynecologists at a tertiary referral center (Oslo University Hospital, Ullevål). The 10 dimensions of the ENDOCARE questionnaire (ECQ) [19] were used as a template to develop the questionnaire in this survey, to ensure to cover relevant items. We added further items specific for the development of an endometriosis center in Norway. The questionnaire was in Norwegian. The questionnaire contained 30 questions with either a 6-point Likert scale (to a very large degree, to a large degree, to some degree, to a small degree, to a very small degree, not at all), or one of two 5-point Likert scales (totally agree, agree, neither agree nor disagree, disagree, totally disagree; Very easy, easy, neither easy nor difficult, difficult, very difficult), additionally 9 multiple choice questions, and 8 open-ended questions. The qualitative part of the survey is based on the answers from 6 out of the 8 open-ended questions that was created for this specific survey.

The living distance from Oslo, which is the capital city and possible location of an endometriosis/adenomyosis center, was gathered as the only background variable.

### Participants and recruitment

Participants were recruited from the NPES. The link to the anonymous digital questionnaire was distributed via the NPES in social media. We used the digital survey tool “Nettskjema” which is operated by the University Information Technology Center at the University of Oslo. The survey was open for everyone with a self-reported diagnosis of endometriosis and/or adenomyosis, their partners and relatives between May 12th and May 16th, 2021. All data were obtained anonymously and no individual could be identified on basis on one response or combinations of responses, and no health data was collected. Informed consent to participate was inferred upon completion of the survey.

### Data analysis

Categorical data were described as frequency (number and percentage). The open-text answers were analyzed following the analytical steps in the Qualitative Content Analysis by Graneheim and Lundman [20]; by open reading, identifying meaning units, creating codes/terms and categories, sorting codes and categories into sub-themes and formulating into latent theme. The first author (MO) and one of the co-authors (TT) was responsible for the analysis. A sample size calculation showed at that at least 384 respondents were recommended for this survey, based on an estimated endometriosis prevalence of 10% amongst women in with a total population of 5 million people in Norway, with a confidence level of 95%, and an alpha of 0.05.

## Results

### Participants

**Fig. 1:**
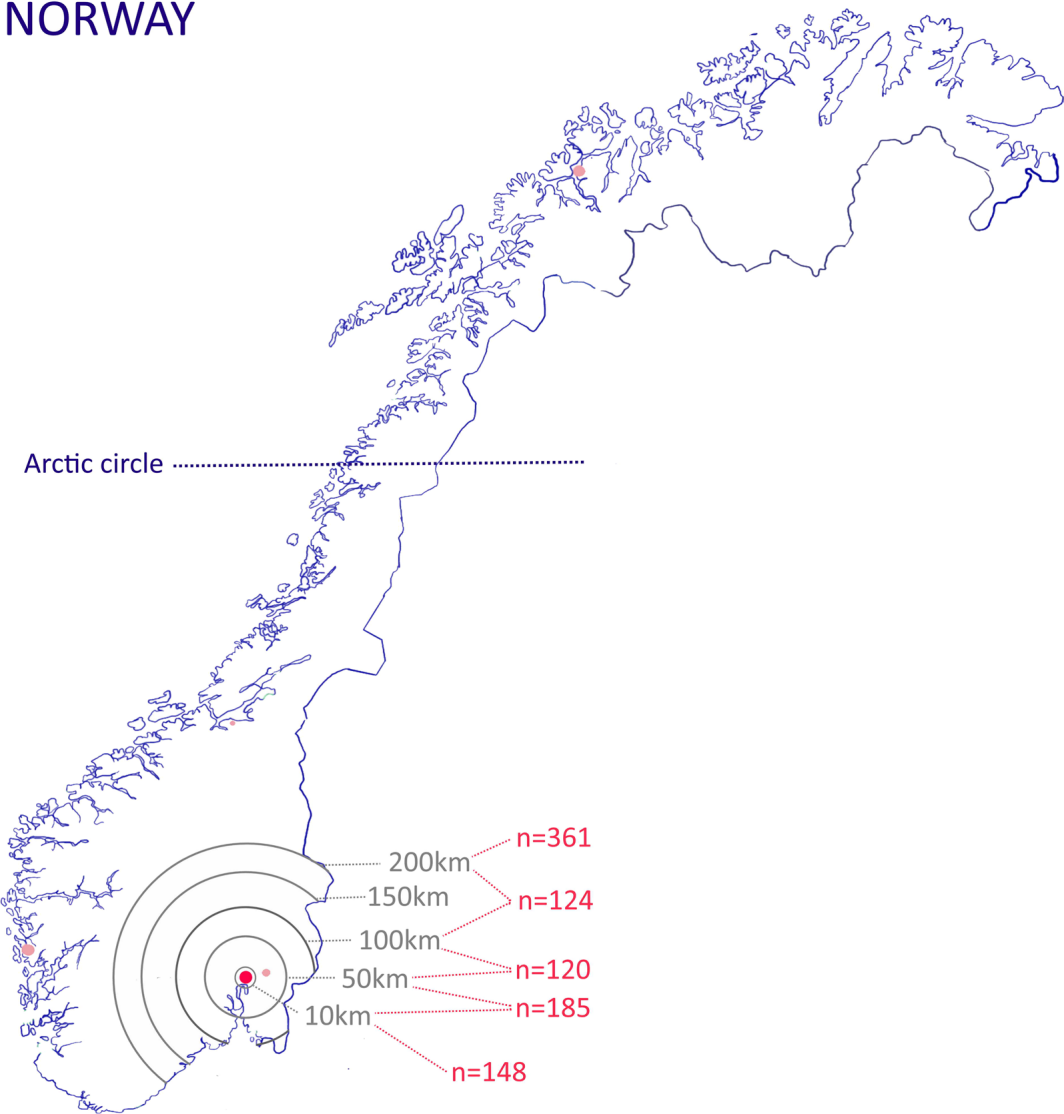
Map illustrating the topography of Norway. Distances from the capital Oslo (red dot) are indicated by grey circles. Number of survey participants from each distance interval are indicated in red. Pink dots indicate the location of the other four university hospitals in Norway

A total of 938 participants answered the questionnaire within 5 days. 873 women with a self-reported diagnosis of endometriosis and/or adenomyosis and 65 relatives participated. The participants’ living distance in relation to the Oslo University Hospital is illustrated in Fig. 1.

### Referral to the endometriosis center

**Fig. 2:**
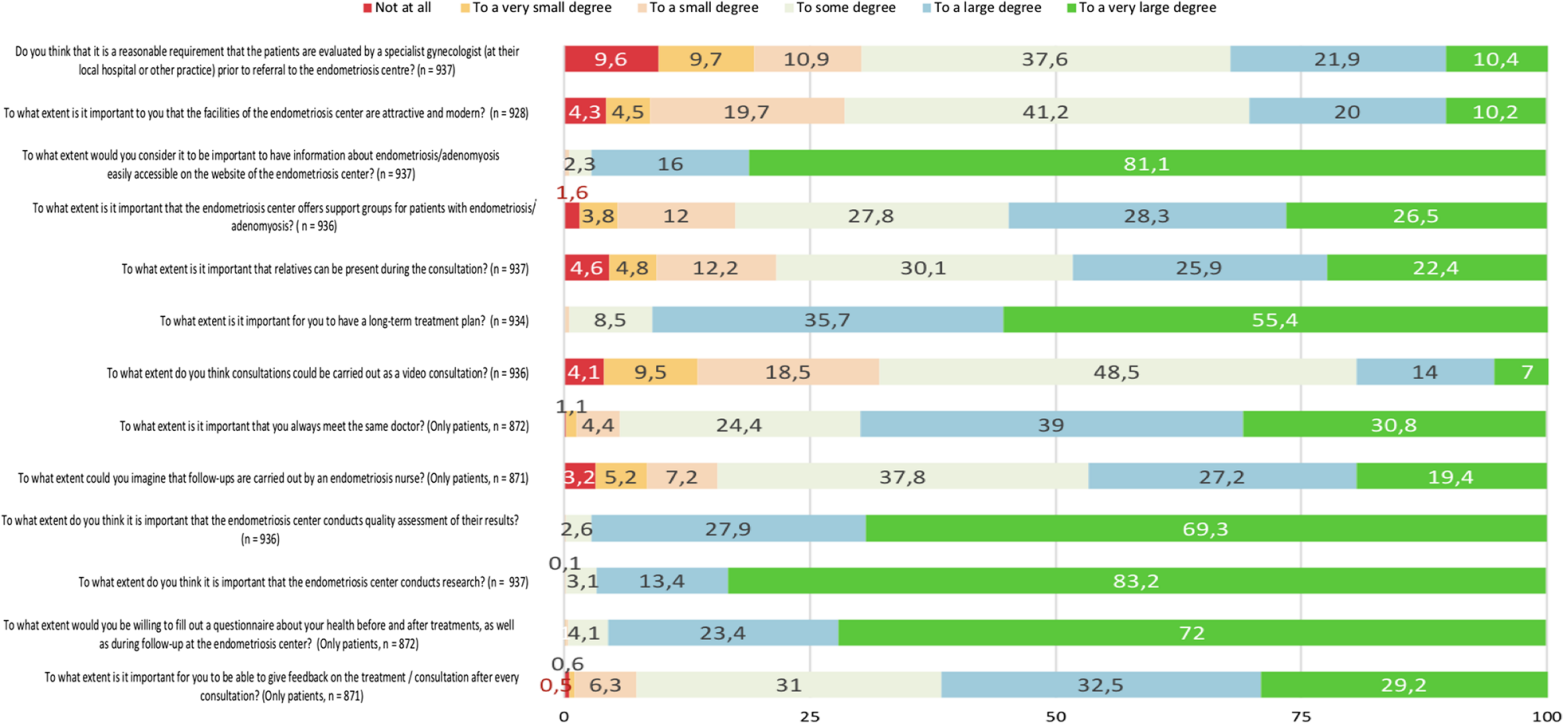
Overview of the results from the questions with a 6-point Likert scale (to a very large degree, to a large degree, to some degree, to a small degree, to a very small degree, not at all) in percentage

The majority of the participants (n = 654; 70%) agreed to some, large or very large degree that it should be a reasonable requirement that the patients are evaluated by a specialist gynecologist (at their local hospital, public or private practice) prior to referral to the endometriosis center (Fig. 2). Two thirds of participants (n = 615; 66%) reported that a waiting time of maximum 6 weeks is acceptable from the first referral from a local hospital to the consultation at the endometriosis center. The percentage increased to 89% (n = 835) when asked about the same acceptable waiting time from referral to consultation for former patients of the endometriosis center (Additional file 1: Table S1).

When asking if an endometriosis center mainly should offer treatment to women with severe endometriosis or adenomyosis, given that resources are limited, only 49% (n = 444) agreed or totally agreed, 18% (n = 166) were neutral and 33% (n = 293) disagreed or totally disagreed. Most of the participants (n = 663; 71%) agreed to some, large or a very large degree that it is of importance that the facilities of the endometriosis center are attractive and modern. The detailed results are displayed in Fig. 2.

### Communication, information and shared decision making

When asked about their preferred way to be informed about endometriosis/adenomyosis (multiple answers possible), the majority of participants favored to receive information orally by the endometriosis specialist (n = 765; 82%), written information was the next most commonly chosen option (n = 653; 70%), followed by information provided by an endometriosis nurse (n = 499; 53%). In the category “other”, that contained the possibility of an open-text answer, only few additional options were suggested by the participants (n = 19; 2%). This included the combination of oral and written information (n = 8), the use of a website (n = 4) and information videos (n = 3). The detailed data are provided in Additional file 1: Table S2.

Virtually all participants (n = 932; 99%) considered it to be important that the center provides relevant information on endometriosis and adenomyosis on their website (Fig. 2).

In an open-ended question, the participants were asked about their thoughts on how they can best be involved in a joint treatment decision. The participants (n = 497) emphasized the importance of their values, preferences and needs being respected, and the importance of knowledgeable health care takers providing them balanced information about endometriosis and adenomyosis, so that they can make a shared decision about treatment. Additional file 1: Table S3 provides the categories and codes with qualitative descriptions.

### Emotional support, involvement of partners and relatives

Most participants (n = 773; 83%) agreed to some degree, a large degree or very large degree that support groups for patients with endometriosis and/or adenomyosis, initiated by the endometriosis center, would be of importance (Fig. 2). When asked about the frequency of meetings with the support group, approximately half of the patients (n = 426; 52%) reported that every third month would be sufficient, while 1 out of 4 (n = 201; 24%) would like to meet up every month (Additional file 1: Table S4).

Most participants (n = 735; 78%) agreed to some degree, a large degree or very large degree that it is of importance that relatives are able to attend the consultation (Fig. 2). A significantly higher percentage of the relatives compared to the patients themselves consider this to be important (n = 61; 94% vs. n = 674; 77%, *p* = 0.001) (Additional file 1: Table S5).

In an open-ended question, that was answered by 269 participants, the participants were asked if they would suggest other ways to involve relatives in the treatment process. Most participants emphasized the importance of providing information to the relatives in general, but a written form or video content was suggested most frequently. Involvement of the partner during the consultations was also a high priority. Support groups for relatives, sex therapy for couples and easily accessible contact information to health care professionals were mentioned as well. The categories and codes for this section topic, with qualitative descriptions are provided in Table 1.

**Table 1:** Results from the open-ended question *“Are there other ways to involve relatives in the treatment process, which should be offered?”.* All codes/terms presented were used by 10 or more participants. Multiple codes/terms could be given per participant

Categories	n	Codes/terms	Representative quotes
Information in general, including all aspects of adenomyosis/endometriosis	206	Information, information in general	*Information and increased knowledge that makes it easier to be supportive as a relative* *A lot more information about the disease in order for relatives to understand what the patients are facing*
Involvement of partner during consultations (with doctor, nurse or endometriosis nurse)	52	Presence of relatives/significant others/partners, involving relatives	*To be able to attend consultations to get information on what I can do as a partner to make things better or more comfortable* *Conversation with a nurse on “how to live with an endo-patient”*
Support groups for relatives	34	Support groups, groups for relatives, courses for relatives	*Groups and information specifically for relatives* *Course/conversation on how to best take care of the patient and the relationship*
Written information tailored to relatives needs	77	Written information/brochure/booklets for relatives	*Information booklets with easily accessible relevant information*
Video information tailored to relatives needs	59	Video/facetime/webinar for relatives	*Information videos meant for relatives* *A basic video about endometriosis to make it easier to understand*
Sex therapy for couples	12	Sex therapy, sexologist	*Option of sex therapy could be helpful since sex life and intimacy may be affected* *Consider sex therapy since talking about intimacy problems is not easy for everyone*
Easily accessible contact information to health care providers	13	Contact, help, ask questions, communication	*The opportunity to talk to healthcare professionals about the situation/patient, be able to ask questions and get all the information you need* *Opportunities for chat communication with a nurse with a short response time*
Other/not classified	17		

### Follow-up and continuity

Nearly all participants emphasized the value of a long-term treatment plan, with the majority considering this to be important to a very large degree (n = 517; 55%), and the remainder to a large degree (n = 333; 36%) or to some degree (n = 79; 8,5%) (Fig. 2). Most participants (n = 636; 68%) agreed to some, large or very large degree that consultations can also be carried out as video consultations (Fig. 2).

Nearly all patients (n = 822; 94%) agreed to some degree, a large degree or very large degree that they wish to meet the same doctor during follow up (Fig. 2). At the same time, most patients (n = 735; 84%) would find it acceptable to some, large or very large degree that follow-ups are carried out by an endometriosis nurse (Fig. 2).

In an open text answer, the patients were asked about what they at the present have missed the most with regards to follow-up and/or treatment. A large proportion of patients (n = 607) reported that they, at present, they did not receive any follow-up at all, consequently this being what they miss/have missed the most. This was especially the case after surgical treatment. Most patients also reported that they did not perceive being taken seriously/believed in/listened to/understood in their dialogue with health care professionals. See Table 2 for detailed information.

**Table 2:** Results from the open-ended question *“What do you miss or have you missed the most with regards to follow-up or treatment as a patient with endometriosis / adenomyosis?”.* All codes/terms presented were used by 10 or more participants. Multiple codes/terms could be given per participant

Categories	n	Codes/terms	Representative quotes
Respect for patients’ values, preferences and needs		To be taken seriously, believed in, listened to, to be met with understanding, respected	*To be believed in and understood.* *Respected. For many years I was told that this was in my head, not in my stomach.* *To be taken seriously. Don’t have track on how many GPs and gynecologists that have sent me home with "No, you must live with this" over the last fifteen years*
In general	186		
To be taken seriously	72		
To be believed in	52		
To be met with understanding, listened to, respected	96		
An earlier diagnosis	51	Diagnosis	*The time it takes to get a diagnosis is so extremely long.* *It took far too long to get a diagnosis. Several years.* *To avoid being a throwing ball for 17 years before anyone could tell me what this was.*
Competence level among health care professionals and in society	153	Competence, knowledge, expertise, society	*More similar knowledge among doctors around the country.* *Knowledge among health professionals. You do not have the strength to be your own advocate when it is at its worst.* *More knowledge in society so that you do not have to suffer in silence and shame*
Information		Information, information on the expected course and prognosis	*I have received very little information about the way forward. Only received the diagnosis during fertility assessment.* *Information about treatment and future opportunities.* *Relatives should be more involved / get information*
In general	112		
Information about the next step and prognosis	27		
Information for relatives	4		
Access to health care services	42	Access, contact point, helpdesk	*The waiting time is too long and there is too little time during consultations. It has also been difficult to get hold of a doctor to ask questions.* *A lower threshold for referral to those who are experts so that you can get proper help*
Multidisciplinary care		Multidisciplinary care, holistic, psychologist, physiotherapist, nutritionist, sexologist	*Really miss follow-up by a multidisciplinary team, both in terms of pain relief, conversations and advice on what you can do yourself to try and get some quality of life.* *A holistic plan for treatment and follow-up. Especially with regards to nutrition, physiotherapy, psychotherapy and medical treatment.* *Follow-up, advice and guidance on what to do. With a focus on coping with everyday life with pain, problems in the relationship, especially due to pain during intercourse, conversations with a partner about being in a relationship with someone with chronic pain*
In general	89		
Psychologist	31		
Physiotherapist	28		
Nutritionist	22		
Sexologist	27		
Follow up		Follow up, follow-up after surgery, continuity, see the same doctor	*Follow-up after treatment and surgery.* *Same doctor who does the follow up.* *Continuity and a feeling of knowing that you can contact competent professionals when needed. An individual plan and a permanent contact person would have been optimal*
In general	271		
After surgery	87		
To see the same doctor	8		
Continuity among health care professionals	45		
Other	18		
Not relevant/not answered the question	26		
Total	1376		

The relatives were also asked about what they perceived as insufficient regarding the follow-up and/or treatment of the patients. They (n = 47) reported the same main areas as the patients themselves; namely planned follow-up and being taken seriously/believed in/listened to/understanded in dialogue with health care professional (Additional file 1: Table S6).

### Multidisciplinary management

**Fig. 3:**
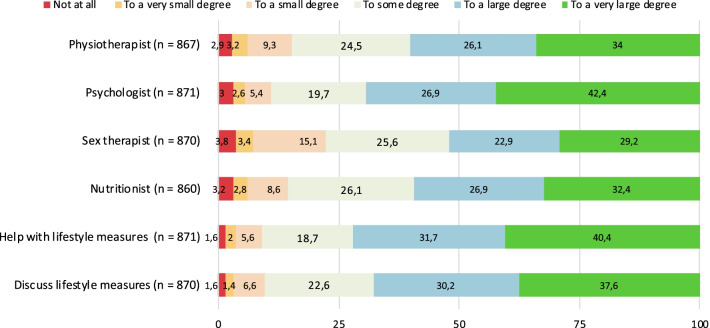
The patients’ need of multidisciplinary care, in percentage

Nearly all patients reported the need for multidisciplinary treatment and follow-up, in terms of the need for a physiotherapist, psychologist, sex therapist, nutritionist, help and support with lifestyle changes and being able to discuss lifestyle measures with a doctor. The detailed data is shown in Fig. 3.

We asked how many consultations with the respective professional (sex therapist, physiotherapist) were considered necessary. Nearly half of the patients reported that just one (n = 372; 44%) or that a few (n = 385; 45%) sessions with a sex therapist would be sufficient to meet their needs. A minority (n = 99; 11.6%) stated to need a more long-term follow-up. Almost half of the patients (n = 397; 46%) reported that they already received physiotherapy for their condition. Most of them (n = 205; 39%) had physiotherapy 1–4 times, while 1 out of 5 (n = 111; 21%) required frequent treatments, with at least 5–10 therapy session. The majority (n = 448; 68%) found it difficult or very difficult to find a physiotherapist with sufficient expertise in treating their condition. See Additional file 1: Table S4 for details of this section.

The participants were asked to provide an open-text answer to what aspects of care they would expect to be offered from an endometriosis center, and which they would as of today not receive from their GP, gynecologist, local hospital or primary health care services. In this open-ended question, the participants answering (n = 614) repeated many previously mentioned needs, such as up to date information, a high competence level among the health care professionals in charge, follow-up and multidisciplinary care. A majority also reported the need for help with pain management. Additional file 1: Table S7 provides the categories and codes with qualitative descriptions.

The participants were asked in what way healthcare professionals could give the patients and their relatives an active role in improving their health. The participants (n = 480) emphasized the importance of showing respect for patients’ values, preferences and needs, knowledge about adenomyosis and endometriosis among health care takers, good communication and easily understandable information about the conditions, multidisciplinary care as an option and a long-term plan for treatment and follow-up. Additional file 1: Table S8 provides the categories and codes with qualitative descriptions.

### Research and quality assessment

All participants found it of importance to some, a large or a very large degree that an endometriosis center quality assures their results (n = 934; 99.8%), and that research is undertaken (n = 935; 99.8%). All patients expressed commitment to contribute to this (n = 868; 99.7%), by filling out questionnaires before and after treatments and during follow up. Patients also reported that being able to give feedback on the consultation after every encounter was of importance to some, a large or a very large degree (n = 807; 93%). See Fig. 2 for details.

## Discussion

In this survey, we identified the patients’ and relatives’ perspectives on best possible care in terms of multidisciplinary management of adenomyosis and endometriosis for potential development of an endometriosis center. The survey also provided new insight on important aspects of patient-centered care, as experienced by this patient group.

One important and central finding of this survey is the great need of more, better provided, better updated and quality assured information for both patients and their relatives. All channels of information provision were considered important, possibly depending on the stage of diagnosis, treatment or follow up. Based on the answers we received, the patients seemed to perceive information as central to enable them to take an active role and adhere to treatment, which is an important factor in shared-decision making and true patient-centered care. These findings are in line with the study by Lukas et al. who found that providing adequate information was highly associated with patient satisfaction [21].

Another important finding was the fact that nearly all participants highlighted the need for a long-term treatment plan. This finding was underlined by the pattern of responses indicating the lack of information about long-term management of endometriosis and the lack of follow-up, especially post-surgery. Chapron et al. stated in their review that it is “time to change the paradigm” about the management of endometriosis. Firstly, endometriosis should be considered as a chronic inflammatory disease and therefore efforts must be made to optimize an individual’s lifelong management plan, focusing more on patients’ symptoms, perspectives and their “endometriosis life” [22].

In the same review the authors stated that “a multidisciplinary approach should be the current standard”, with specialized referral centers being the gold standard [22]. The responses in our survey confirm the need for a multidisciplinary approach, inclusive of a physiotherapist, psychologist, nutritionist, sex therapist and help with necessary lifestyle changes. Timely diagnosis, competent endometriosis health care professionals and up-to-date information was also emphasized. Lack of these parameters are likely to alter the optimal management of these patients. On one hand, employing multiple disciplines is costly and therefore, not feasible for many units. On the other hand, it was highlighted by the patients that in many cases a single, or few consultations would have a relevant impact on their care. A reorganization and effectivization of care could possibly free some resources without additional costs to be able to cater to relevant aspects of patient needs. Other measures, such as patient support groups, demand a relatively low grade of resources with a potential great impact, and more effort to organize and investigate the relevance of this should be undertaken. The fact that about two thirds of the participants agreed that consultations also can be carried out as video consultations, is a particular interesting finding in this regard. The use of video consultations could aid overcoming the issue of the limited resources, interfering with the development of a multidisciplinary approach. As an example, psychological consultations and support group meetings could be organized online, and it could also facilitate doctor continuity during follow-up.

An important aspect of a multidisciplinary center is having the clinical expertise, but to also lead research in the field [14]. Nearly all participants highlighted the importance of the endometriosis center conducting research and to quality assure their results. The survey indicates that patients are more than willing to fill out questionnaires about their health before and after treatments and during follow-up, which can be used to evaluate improvement in patient-centered care and endometriosis management. These findings are reassuring, however, we did not explore the willingness to participate in randomized controlled trials which are highly important but can struggle to recruit endometriosis patients [23].

Another important finding was the responses indicating the lack of integrated care that secures continuity between different health care professionals, especially before getting a diagnosis and after surgery. Many patients feel that they are left on their own, without easily accessible help from competent health care providers. Acknowledging psychological distress and supporting women in handling their symptoms not only positively affects patient satisfaction with medical support [21], but should be considered an indispensable part of treatment. A multidisciplinary referral center should systematically pursue the psychological support of women with endometriosis through listening, explanation, and reassurance. All too often women are scared regarding their overall prognosis, including pain symptoms, fertility, and type of surgical procedures needed.

As endometriosis and adenomyosis are very common, a single, multidisciplinary center can’t treat all patients with endometriosis and/or adenomyosis. Therefore, efforts must be made to strengthen the overall competence level for management of endometriosis and adenomyosis nationwide. This can be facilitated by developing interdisciplinary communication and integrated patient management between an endometriosis center and referring health care professionals.

Although the primary aim of this survey was to describe perspectives on best possible care in terms of developing a multidisciplinary center for adenomyosis and endometriosis, the responses in this survey indicate that endometriosis and adenomyosis patients need more than effective and safe care. A main focus area emerged and should receive special attention, as respecting and believing in patients, which in the ECQ is labeled under “respect for patients’ values, preferences and needs” [17], seems to be an unmet need. This finding is also coherent with a systematic review on patient-centeredness in endometriosis care [24]. However, this seems also to be valid for other chronic diseases. In a qualitative study interviewing stakeholders within rheumatoid arthritis, Barber et al. [25] made similar findings with regards to needs and preferences, where multidisciplinary care and patient respect were central. Patient respect should be a cornerstone of medical practice in general and specific training of health care professionals caring for that patient group should be consequently prioritized.

For future research, it would also be relevant to investigate possible discrepancies between self-perceived patient centeredness and beliefs amongst health care professionals, and to investigate and improve quality of care markers. A possible quality of care marker could be measurement of “the burden of treatment” [26]. For patients with endometriosis this could for example represent care that avoids excessive or useless diagnostic and therapeutic procedures, reserving the least tolerated and most expensive drugs only for non-responders to first-line medications, planning cost-effective and reasonable follow-up, and so on [27]. In our opinion, a multidisciplinary referral center for endometriosis is much more suited to provide this for endometriosis patients, than non-organized care. However, reducing the burden of treatment needs to be a central aim also for endometriosis centres and while this aspect has not been included in our survey, we suggest investigating this in future studies of this type.

The survey has several limitations, including lack of demographic data which could have supplemented both the quantitative and qualitative data. The survey was administered through the Norwegian Patient’s Endometriosis Society through an open, anonymous questionnaire, and there’s no control of specific diagnosis of either endometriosis or adenomyosis among the patients. NPES had 607 members at the time the survey was conducted, which is less than the number of patients participating in the survey (n = 938). NPES has over the last couple of years been an active group and they are currently increasing in numbers, and just passed 1000 members in October 2021.

A strength of this survey, especially in the context of Norway, is the high number of participants and its’ originality with the supplement of qualitative approach with open-text answers. A question is if the results of this national survey are transferrable to other countries and cultural context. We believe that this is the case, as the symptoms and challenges described by people with endometriosis, as expressed through questionnaires and quality of life tools, were consistently valid internationally [28, 29].

The survey is based on the ECQ, an endometriosis-specific and validated questionnaire, which strengthens the relevance of the collected data [24]. As for now, an adenomyosis-specific questionnaire does not exist, and it might be that people with adenomyosis have other, unmet needs. However, in our experience, the overlap in prevalence and symptoms between the conditions is large.

Our survey provides a unique insight into the patients’ and relatives’ perspectives on best possible care of endometriosis management, in a population, whereas of today, a centralized, multidisciplinary endometriosis center does not yet exist. When developing such a center, efforts must be done to include patient-centeredness as a parameter of quality of care, to continuously improve health care services for this patient group nationwide and also for being able to learn from other endometriosis clinics outside Norway.

## Conclusion/recommendation

The results from this survey and the highlighted aspects of patient-centered care for this population, should be weighted in planning and development of a multidisciplinary center. They should also be taken into consideration outside of specialist centers, by health care professionals who encounter this patient group in order to improve continuity of care. These perspectives are only studied in a small number of studies for this patient group and further studies on patient-centered care in endometriosis and adenomyosis care are warranted.

## Supplementary Information

**Additional file 1.** Supplementary tables with results from questions about waiting time from referral, involvement of relatives, information types and the need for consultations with a sex therapist and a physiotherapist, and results from open-text questions about how to give patients and relatives an active role, involvement in a joint treatment decision, what patients and their relatives have missed the most with regards to follow-up or treatment and expectations to an endometriosis center.

**Additional file 2.** Survey "The users' perspectives on best possible care in the context of developing a center for endometriosis and adenomyosis". English version.

## Data Availability

A translated copy of the survey in English is provided in Additional file 2. All data generated or analyzed during this study are included in this published article. The data that support the findings of this survey are available from the authors upon reasonable request, except the individual responses to the open questions.

## Abbreviations

ECQ: ENDOCARE questionnaire
NPES: Norwegian patient’s endometriosis society
