# Supplementary material for: Patients’ and relatives’ perspectives on best possible care in the context of developing a multidisciplinary center for endometriosis and adenomyosis: findings from a national survey
Source: BMC Womens Health. 2022 Jun 10;22:219. doi: 10.1186/s12905-022-01798-8 (PMC9188072; doi:10.1186/s12905-022-01798-8)
Supplement: Supplementary file 1 — Additional file 1. Supplementary tables with results from questions about waiting time from referral, involvement of relatives, information types and the need for consultations with a sex therapist and a physiotherapist, and results from open-text questions about how to give patients and relatives an active role, involvement in a joint treatment decision, what patients and their relatives have missed the most with regards to follow-up or treatment and expectations to an endometriosis center. [file 12905_2022_1798_MOESM1_ESM.pdf]

Supplementary Table 1

	2 weeks	4 weeks	6 weeks	2 months	3 months	4 months	4-6 months	Total
Question A: "What do you consider an acceptable waiting time from a re-referral until the consultation if you are a former patient at the endometriosis center?"								
Patients	74	246	244	186	93	17	11	871
Relatives	17	23	11	11	3	0	1	66
**Total**	**91 (9,7%)**	**269 (28,7%)**	**255 (27,2)**	**197 (21%)**	**96 (10,2%)**	**17 (1,8%)**	**12 (1,3%)**	**937**
Question B. "What do you consider an acceptable waiting time from the first referral from a local hospital/gynecologist until the consultation at the endometriosis center for newly referred patients?"								
Patients	244	351	182	64	21	3	5	870
Relatives	20	26	12	7	0	0	0	65
**Total**	**264 (28,2)**	**377 (40,3)**	**194 (20,7%)**	**71 (7,7%)**	**21 (2,2%)**	**3 (0,3%)**	**5 (0,5%)**	**935**

Supplementary Table 1: Time frames from referral to consultation, patient and relatives responses.

Supplementary Table 2

"In what way do you prefer to be informed about endometriosis and/or adenomyosis?". Multiple answers available, therefore the sum is greater than the total number of participants.					
	**Orally by the gynecologist specialist**	**Orally by the endometriosis nurse**	**Written information**	**Other**	**Total**
Patients	720	464	606	18	1808
Relatives	45	35	47	1	128
**Total**	**765**	**499**	**653**	**19**	**1936**

Supplementary Table 2: Response rates, information dissemination.

Supplementary Table 3

**Categories**	**n**	**Codes/terms**	**Representative quotes**
Respect for patients' values, preferences and needs	318	To be taken seriously, believed in, listened to, to be met with understanding, respected, included	*To be informed about the possibilities when it comes to surgery and medications, and then be an active part in choosing what is best for me.*<br><br>*To be listened to.*<br><br>*Through questionnaires like this one. It's great that patients can be listened to.*
Coordination and integration of care, multidisciplinary care	94	Continuity between health care professionals, multidisciplinary care, holistic care, coordination	*Multidisciplinary collaboration between doctor, physiotherapist, gynecologist, patient.*<br><br>*Having a coordinator who talks to various therapists when needed, helps me keep track of status and keep track of the type of treatment that gives an improvement or not.*
General information and advice, communication and education	223	General information, advice, lifestyle measures, competence,	*More time during consultations.*<br><br>*Dialogue instead of monologue.*
Emotional support	22	Support from relatives/family, support groups,	*Conversations with other patients.*

		motivation, Patients' Endometriosis Society	
Involvement of significant others	17	Relatives/significant others	*That those around me also can express how my life is affected.*<br><br>*A decision regarding treatment should preferably be made by the specialists and the patient, but that I as a relative can be involved and be informed about the plan, possible complications and the time after a treatment.*
Access to care, plan for treatment	34	Access to care, plan for treatment, follow-up	*Options for easier access to health care takers*
Other	39		
Total	747		

Supplementary Table 3: Results from the open-ended question "*In your opinion, what way would be the best for you to be involved in a joint treatment decision*?". Responses only from patients. All codes/terms presented were used by 10 or more participants. Multiple codes/terms could be given per participant.

Supplementary Table 4

Q: How often would you like to have these meetings with other patients with endometriosis / adenomyosis?

	Weekly	Every 2 weeks	Every 3 weeks	Every month	Every 2 months	Every 3 months	Total
Patients (n/%)	6 (0,7%)	21 (2,5%)	24 (2,9%)	201 (24,3%)	148 (17,9%	426 (51,6%)	826

Q: If you had been offered a conversation with a sexologist, which of the following offers would have been relevant for you?

	One consultation	A few consultations	Long-term	Patients
Patients (n/%)	372 (43,5%)	385 (45%)	99 (11,6%)	856

Q: Have you previously had physiotherapy due to symptoms caused by or associated with endometriosis, adenomyosis or pelvic pain?

	Yes	No	Patients
Patients (n/%)	397 (45,5%)	475 (54,5%)	872

Q: How easy/difficult was it to find a physiotherapist which you perceived had expertise in your condition?

	Very easy	Easy	Neither easy or difficult	Difficult	Very difficult	Total
Patients (n/%)	12 (1,8%)	31 (4,7%)	167 (25,4%)	136 (20,7%)	312 (47,4%)	658

Q: How many times have you seen the physiotherapist for treatment?

	One time	2-4 times	5-10 times	10-20 times	20-30 times	Over 30 times	Total
Patients (n/%))	104 (19,8%)	101 (19,2%)	111 (21,1%)	79 (15%)	38 (7,2%)	93 (17,7%)	826

Supplementary Table 4: Results from the questions about frequency of meeting other health care professionals and patient groups.

Supplementary Table 5

"To what extent is it important that relatives can be present during the consultation?"

	To a very large extent	Largely	To some degree	To a small degree	To a very small extent	Not at all	Total
Patients	193 (22,1%)	220 (25,2%)	261 (30%)	43 (4,9%)	113 (12,9%)	42 (4,9%)	872
Relatives	17 (26,1%)	23 (35,4%)	21 (32,3%)	2 (3,1%)	1 (1,5%)	1 (1,5%)	65
**Total**	**210**	**243**	**282**	**45**	**114**	**43**	**937**

Supplementary Table 5: Responses regarding relative participation.

Supplementary Table 6

Categories	n	Codes/terms	Representative quotes
Respect for patients' values, preferences and needs	13	To be taken seriously, believed in, listened to, to be met with understanding, respected	*I know that those affected feel a sense of hopelessness over the lack of treatment and talking to deaf ears.*
An earlier diagnosis	2	Diagnosis	
Competence level among health care professionals and in society	11	Competence, knowledge, expertise, society	*Knowledge among GPs.*
Information	11	Information, information on the expected course and prognosis	*The risk of infertility applies to more than just the person with endometriosis / adenomyosis. It can be helpful to talk about it under the guidance of a healthcare professional.*
Access to health care services	5	Access, contact point, helpdesk	
Interdisciplinary care	2	Interdisciplinary care, holistic, psychologist, physiotherapist, nutritionist, sexologist	
Follow up	16	Follow up, follow-up after surgery, continuity, see the same doctor	*Follow-up is currently totally absent, unfortunately.*
Other (items < 5)	1		
Total	61		

Supplementary Table 6: Results from the open-ended question "What do you miss or have you missed the most as a relative to someone with endometriosis/adenomyosis, with regards to follow-up or treatment?". Responses from relatives only. Representative quotes are given for the categories where the codes/terms are used by 10 or more participants. Multiple codes/terms could be given per participant.

Supplementary Table 7

Categories	n	Codes/terms	Representative quotes
Respect for patients' values, preferences and needs	127	To be taken seriously, believed in, listened to, to be met with understanding, respected	*To be listened to and believed is as important as treatment.*<br><br>*To be believed in and understood early on.*
An earlier diagnosis	17	Diagnosis	*That you can avoid having to be tormented for over ten years by other health professionals who do not have experience or knowledge on the subject.*
Competence level among health care professionals and in society	165	Competence, knowledge, expertise, knowledge in society, evidence-based	*Training of doctors at the local hospitals, emergency services and gynecologists. Not everyone needs to go to the endometriosis center, if we are met with understanding and a minimum of knowledge.*<br><br>*Meet health care providers with evidence-based knowledge and stay professionally up to date.*
Information	114	Information about the disease, information on the expected course and prognosis	*Thorough and good information.*<br><br>*Offer to understand the course of the disease and good information.*
Access to health care services	27	Access, availability, contact	*Easier to achieve contact with the attending physician, without having to be referred again from the GP when one has already been diagnosed and treated / operated on for endo.*
Multidisciplinary care<br>- In general<br>- Psychologist<br>- Physiotherapist<br>- Nutritionist<br>- Sexologist	 78<br>33<br>34<br>22<br>16	Multidisciplinary care, holistic, psychologist, physiotherapist, nutritionist, sexologist	*Multidisciplinary treatment by people specialized in endometriosis.*<br><br>*Treatment, pain management, psychologist, nutrition.*
Pain management	76	Pain management, pain relief, pain specialist, pain killers	*Help to make a plan for pain management and what to do when the endometriosis gets worse.*<br><br>*Help to cope with everyday life with a lot of pain. Help to know what to do if you do not cope with the pain yourself.*
Follow up	191	Follow up in general, follow-up after surgery, continuity, see the same doctor	*Follow-up and information. A plan for treatment knowing based on the fact that it is a chronic disease.*<br><br>*Follow-up and help to improve daily life. That the patient doesn't "fall out" of the system after surgery.*
Coordination between GP, gynecologist, local hospital and endometriosis center	22	Coordination	*It is important to have the opportunity for the GP to collaborate with a specialist in relation to medication and possibly other treatment.*
Assessment and treatment	52	Assessment, treatment, ultrasound, MRI, advanced surgery	*Professional conversation, examination, MRI and surgery.*
Conducts research	15	Research, science	*Opportunity to participate in research projects.*
Not relevant/not answered the question	43		
Total	958		

Supplementary Table 7: Results from the open-ended question "*What services would you expect to receive from an endometriosis center, which you as a patient/relative do not receive from your GP / gynecologist / local hospital / primary health service)*?". All codes/terms presented were used by 10 or more participants. Multiple codes/terms could be given per participant.

Supplementary Table 8

Categories	n	Codes/terms	Representative quotes
Respect for patients' values, preferences and needs	200	Respect, to be taken seriously, listened to, empathy, believed in included	*Listen to what I have to say.*<br><br>*Treat us as human beings and individuals, and not just as an object.*
Coordination and integration of care, multidisciplinary care	49	Continuity between health care professionals, multidisciplinary care, holistic care, coordination	*Ensure an interdisciplinary care so that all the different aspects of the disease are addressed, then it will be easier to make relevant changes.*<br><br>*It would have been nice to have an interdisciplinary plan where all parties know their role.*
General information and advice, communication and education	261	General information, advice, lifestyle measures, competence,	*Better information about what is happening will provide increased security and peace of mind, both for the patient and relatives.*<br><br>*Give me information on what I can do. I want to make lifestyle changes etc., but I need to know what I should do.*<br><br>*The person in question must have knowledge and competence.*
Emotional support	36	Support from relatives/family, support groups, motivation, Patients' Endometriosis Society	*We must all take responsibility, no therapist can give us advice if we are not willing to listen, but the therapist can be an inspirer to actively take responsibility for our own health.*<br><br>*Give me as a patient tools to deal with the disease*
Involvement of relatives/significant others	14	Relatives/significant others	*Information to relatives about everyday problems and create a better understanding with relatives*<br><br>*Support system and info for relatives.*
Access to care, plan for treatment	106	Access to care, plan for treatment, follow-up	*By receiving regular follow-up and not just being left to myself.*<br><br>*Be available during treatment.*
Other	31		
Total	697		

Supplementary Table 8: Results from the open-ended question "*In what way can a healthcare professional give you an active role in improving your own health, or to you as a relative of someone with endometriosis/adenomyosis*?" All codes/terms presented were used by 10 or more participants. Multiple codes/terms could be given per participant
