# Supplementary material for: Patients’ and relatives’ perspectives on best possible care in the context of developing a multidisciplinary center for endometriosis and adenomyosis: findings from a national survey
Source: BMC Womens Health. 2022 Jun 10;22:219. doi: 10.1186/s12905-022-01798-8 (PMC9188072; doi:10.1186/s12905-022-01798-8)
Supplement: Supplementary file 2 — Additional file 2. Survey "The users' perspectives on best possible care in the context of developing a center for endometriosis and adenomyosis". English version. [file 12905_2022_1798_MOESM2_ESM.pdf]

The users' perspectives on best possible care in the context of developing a center for endometriosis and adenomyosis

Page 1

Mandatory fields are marked with a star *

We at the Department of Gynecology at Ullevål Hospital want to improve our service for women with endometriosis and adenomyosis. In order to develope a tailored health service and possible center for endometriosis and adenomyosis based on the patients' needs, we need to know what is important to you and your relatives.

We want to get as much input as possible from women with endometriosis / adenomyosis and their relatives, and we are grateful that you take the time to answer this survey. We have some specific questions at the beginning, but you will have the opportunity to write free text answers at the end.

It is important to emphasize that the Department of Gynecology at Ullevål Hospital has not been allocated funds for the development of an endometriosis/adenomyosis center. Also, we have not received any signals from the government that it is planned to allocate us earmarked funds. Therefore, all activity around an endometriosis center must be funded within the existing framework of resources we have per today. It is therefore important to prioritze services offered based on your feedback.

In this survey, while we ask what your vision / ideal situation for an endometriosis center would be, we want to know as well what you find most important and what should be prioritized, in your opinion. This questionnaire is anonymous. Please do not enter sensitive personal data in the open fields.

Background info

Please check the appropriate box, are you a: *

- ○ Patient (diagnosed with endometriosis and/or adenomyosis)
- ○ Relative

How far away from Ullevål Hospital do you live?

- ☐ 0-10km
- ☐ 10-20km
- ☐ 20-50km
- ☐ 50-100km
- ☐ 100-200km
- ☐ Over 200km

Page break

Page 2

Mandatory fields are marked with a star *

Referral to the endometriosis center

Please answer the questions based on your own needs, not what you assume applies to patients you know.

Do you think that it is a reasonable requirement that the patients are evaluated by a specialist gynecologist (at their local hospital or other practice) prior to referral to the endometriosis center?

- ◯ To a very large degree
- ◯ To a large degree
- ◯ To some degree
- ◯ To a small degree
- ◯ To a very small degree
- ◯ Not at all

What do you consider an acceptable waiting time from the first referral from a local hospital/gynecologist until the consultation at the endometriosis center for newly referred patients?

- ☐ 2 weeks
- ☐ 4 weeks
- ☐ 6 weeks
- ☐ 2 months
- ☐ 3 months
- ☐ 4 months
- ☐ 4-6 months

What do you consider an acceptable waiting time from a re-referral until the consultation if you are a former patient at the endometriosis center?

- ☐ 2 weeks
- ☐ 4 weeks
- ☐ 6 weeks
- ☐ 2 months
- ☐ 3 months
- ☐ 4 months
- ☐ 4-6 months

How much do you agree with this statement: In light of the lack of resources, should the focus of the endometriosis center be on women with high-grade endometriosis / adenomyosis?

- ☐ Totally agree
- ☐ Agree
- ☐ Neither agreee or disagree
- ☐ Disagree
- ☐ Totally disagree

Page break

Page 3

Mandatory fields are marked with a star *

Follow-up

To what extent do you think consultations could be carried out as a video consultation?

- To a very large degree
- To a large degree
- To some degree
- To a small degree
- To a very small degree
- Not at all

To what extent could you imagine that follow-ups are carried out by an endometriosis nurse?

- To a very large degree
- To a large degree
- To some degree
- To a small degree
- To a very small degree
- Not at all

To what extent is it important that you always meet the same doctor?

- To a very large degree
- To a large degree
- To some degree
- To a small degree
- To a very small degree
- Not at all

To what extent is it important that relatives can be present during the consultation?

- To a very large degree
- To a large degree
- To some degree
- To a small degree
- To a very small degree
- Not at all

Are there other ways to involve relatives in the treatment process, which should be offered?

To what extent is it important for you to have a long-term treatment plan?

- To a very large degree
- To a large degree
- To some degree
- To a small degree
- To a very small degree
- Not at all

Page break

Page 4

Mandatory fields are marked with a star *

Research and quality assessment

To what extent do you think it is important that the endometriosis center conducts quality assessment of their results?

- To a very large degree
- To a large degree
- To some degree
- To a small degree
- To a very small degree
- Not at all

To what extent do you think it is important that the endometriosis center conducts research?

- To a very large degree
- To a large degree
- To some degree
- To a small degree
- To a very small degree
- Not at all

To what extent would you be willing to fill out a questionnaire about your health before and after treatments, as well as during follow-up at the endometriosis center?

- To a very large degree
- To a large degree
- To some degree
- To a small degree
- To a very small degree
- Not at all

To what extent is it important for you to be able to give feedback on the treatment / consultation after every consultation?

- To a very large degree
- To a large degree
- To some degree
- To a small degree
- To a very small degree
- Not at all

Page break

Page 5

Mandatory fields are marked with a star *

Information and communication

In what way do you prefer to be informed about endometriosis and/or adenomyosis?

- Orally by the gynecologist specialist
- Orally by the endometriosis nurse
- Written information
- Other

To what extent would you consider it to be important to have information about endometriosis /adenomyosis easily accessible on the website of the endometriosis center?

- To a very large degree
- To a large degree
- To some degree
- To a small degree
- To a very small degree
- Not at all

In your opinion, who has the main responsibility for informing other healthcare professionals about endometriosis / adenomyosis?

	To a very large degree	To a large degree	To some degree	To a small degree	To a very small degree	Not at all
The Endometriosis Association (Patient Organization)	☐	☐	☐	☐	☐	☐
Endometriosis Center	☐	☐	☐	☐	☐	☐
The Norwegian Medical Association	☐	☐	☐	☐	☐	☐
The Norwegian Directorate of Health	☐	☐	☐	☐	☐	☐
University (teaching of medical students)	☐	☐	☐	☐	☐	☐

Page break

Page 6

Mandatory fields are marked with a star *

Expectations towards the endometriosis center

What services would you expect to receive from an endometriosis center, which you as a patient/relative do not receive from your GP / gynecologist / local hospital / primary health service)?

To what extent is it important to you that the facilities of the endometriosis center are attractive and modern?

- ○ To a very large degree
- ○ To a large degree
- ○ To some degree
- ○ To a small degree
- ○ To a very small degree
- ○ Not at all

To what extent is it important that the endometriosis center offers support groups for patients with endometriosis/adenomyosis?

- ○ To a very large degree
- ○ To a large degree
- ○ To some degree
- ○ To a small degree
- ○ To a very small degree
- ○ Not at all

How often would you like to have these meetings with other patients with endometriosis / adenomyosis?

ℹ This element is only shown when the option "Patient (diagnosed with endometriosis and/or adenomyosis)" is selected in the question "Please check the appropriate box, are you a:"

- ○ Weekly
- ○ Every 2 weeks
- ○ Every 3 weeks
- ○ Every month
- ○ Every 2 months
- ○ Every 3 months

Page break

Page 7

Mandatory fields are marked with a star *

Multidisciplinary treatment

To what extent would you have the need/would it be important for you to have a consultation with a nutritionist?

- To a very large degree
- To a large degree
- To some degree
- To a small degree
- To a very small degree
- Not at all

To what extent is it important for you to discuss lifestyle measures / advice with a doctor?

- To a very large degree
- To a large degree
- To some degree
- To a small degree
- To a very small degree
- Not at all

This element is only shown when the option "Patient (diagnosed with endometriosis and/or adenomyosis)" is selected in the question "Please check the appropriate box, are you a:"

In your opinion, what kind of healthcare professionals are best suited to discuss lifestyle advice with you?

	To a very large degree	To a large degree	To some degree	To a small degree	To a very small degree	Not at all
Gynecologist	☐	☐	☐	☐	☐	☐
Endometriosis nurse	☐	☐	☐	☐	☐	☐
General practitioner	☐	☐	☐	☐	☐	☐
Nutritionist	☐	☐	☐	☐	☐	☐

Several women experience different challenges living with endometriosis / adenomyosis. To what degree do you feel that you could benefit from a mapping consultation with a psychologist as part of your treatment?

- ○ To a very large degree
- ○ To a large degree
- ○ To some degree
- ○ To a small degree
- ○ To a very small degree
- ○ Not at all

Some women with endometriosis / adenomyosis experience that everything becomes "too much" and that they don't manage to make the necessary lifestyle changes. To what extent would a program that supports you implementing lifestyle changes be useful to you?

- ○ To a very large degree
- ○ To a large degree
- ○ To some degree
- ○ To a small degree
- ○ To a very small degree
- ○ Not at all

To what extent would it be important for you to get a consultation with a sex therapist?

- ○ To a very large degree
- ○ To a large degree
- ○ To some degree
- ○ To a small degree
- ○ To a very small degree
- ○ Not at all

If consultations with a sex therapist would be offered, which of the following would be the right option for you?

ⓘ This element is only shown when the option "Patient (diagnosed with endometriosis and/or adenomyosis)" is selected in the question "Please check the appropriate box, are you a:"

- ○ One consultation would be enough
- ○ I would have needed a few consultations
- ○ I would have needed a more long-term offer

To what extent is it important for you to have physiotherapy as part of your treatment?

- To a very large degree
- To a large degree
- To some degree
- To a small degree
- To a very small degree
- Not at all

Have you previously had physiotherapy treatment due to symptoms caused by or associated with endometriosis, adenomyosis or pelvic pain?

This element is only shown when the option "Patient (diagnosed with endometriosis and/or adenomyosis)" is selected in the question "Please check the appropriate box, are you a:"

- Yes
- No

How easy/difficult was it to find a physiotherapist which you perceived had expertise in your condition?

This element is only shown when the option "Patient (diagnosed with endometriosis and/or adenomyosis)" is selected in the question "Please check the appropriate box, are you a:"

- Very easy
- Easy
- Neither easy or difficult
- Difficult
- Very difficult

How many times have you seen the physiotherapist for treatment?

This element is only shown when the option "Patient (diagnosed with endometriosis and/or adenomyosis)" is selected in the question "Please check the appropriate box, are you a:"

- One time
- 2-4 times
- 5-10 times
- 10-20 times
- 20-30 times
- More than 30 times

How far away from your home is the physiotherapy practice?

This element is only shown when the option "Patient (diagnosed with endometriosis and/or adenomyosis)" is selected in the question "Please check the appropriate box, are you a:"

Page break

Page 8

Mandatory fields are marked with a star *

Your thoughts on follow-up / treatment

Finally, we are asking you to answer three open-ended questions about your follow-up and treatment. Please do not write sensitive personal data.

In your opinion, what way would be the best for you to be involved in a joint treatment decision?

In what way can a healthcare professional give you an active role in improving your own health, or to you as a relative of someone with endometriosis/adenomyosis?

What do you miss or have you missed the most with regards to follow-up or treatment of you as a patient with endometriosis / adenomyosis?

This element is only shown when the option "Patient (diagnosed with endometriosis and/or adenomyosis)" is selected in the question "Please check the appropriate box, are you a:"

What do you miss or have you missed the most as a relative to someone with endometriosis / adenomyosis with regards to follow-up or treatment?

This element is only shown when the option "Relative" is selected in the question "Please check the appropriate box, are you a:"

[See recent changes in Nettskj](https://nettskjema.no)
